# In silico mining of microsatellites from the Phlebotomus argentipes (Diptera: Psychodidae) scaffold-level genome assembly and validation of eleven genotyping markers

**DOI:** 10.21203/rs.3.rs-9513723/v1

**Published:** 2026-06-08

**Authors:** Navoda Wijesuriya, Sachee Bhanu Piyasiri, Vidyani Kulatunga, B.G.D. Nissanka K. de Silva, Nadira Karunaweera

**Affiliations:** University of Colombo; University of Colombo; University of Colombo; University of Sri Jayewardenepura; University of Colombo

## Abstract

*Phlebotomus argentipes* is the primary vector of *Leishmania donovani*, the causative agent of leishmaniasis. This study aimed to expand genomic resources for *P. argentipes* by *in silico* mining and characterization of simple sequence repeats (SSRs) from a scaffold-level genome assembly retrieved from GenBank, followed by wet-lab validation of selected SSR markers. A total of 22 SSR loci were identified from intergenic regions and used for primer design. Laboratory validation using PCR amplification of genomic DNA from laboratory-reared flies confirmed successful amplification across all loci. Based on amplification quality and verification of SSR motifs, 11 primer pairs were selected for fragment analysis and allelic characterization in field-collected *P. argentipes* (n = 20) from Ambalantota (Geo 1) and Polpithigama (Geo 2). All selected loci exhibited polymorphism, with allelic diversity ranging from 1 to 8 alleles per locus. Observed heterozygosity (Ho) ranged from 0.00 to 0.75, expected heterozygosity (He) from 0.29–0.85, and polymorphism information content (PIC) from 0.26–0.81, with four markers (IC27, IC51, IC68, IC74). Genetic differentiation between populations (F_ST_) ranged from 0.03–0.10, while F_IT_ ranged from 0.05–0.45, indicating heterozygote deficit across loci, except for IC21, which showed heterozygote excess, supporting the utility of these SSR markers for population genetic studies.

## Introduction

Leishmaniasis is a group of diseases caused by protozoan parasites belonging to over 20 *Leishmania* species and transmitted by more than 90 sand fly species worldwide [1]. There are three main clinical forms of the disease: cutaneous leishmaniasis (CL), mucocutaneous leishmaniasis (MCL) and visceral leishmaniasis (VL), also known as kala-azar. The World Health Organization (WHO) identifies CL as the most prevalent form of the disease, VL as the most severe, and MCL as the most disabling [1]. CL is an emerging, uncontrolled and neglected infection, affecting millions each year [1]. Meanwhile, VL is considered the second-most deadly parasitic disease in tropical and subtropical regions, posing a significant public health challenge [1].

The sand fly, *Phlebotomus argentipes* (Diptera: Psychodidae), is the incriminated vector of *Leishmania donovani*, the causative agent of leishmaniasis in Sri Lanka, and other Asian countries [2–6]. Effective control of *P. argentipes* populations is crucial for reducing Leishmania transmission, emphasizing the need for robust vector control strategies, including insecticide-based interventions, environmental management, and community awareness programs.

Furthermore, genetic studies of *P. argentipes* are essential for understanding population dynamics, insecticide resistance, and vector competence, thereby informing more targeted and sustainable control efforts. Integrating genetic research on the parasite and its vector into disease control programs could enhance disease surveillance, improve intervention strategies, and contribute to the long-term elimination of the disease.

Simple sequence repeats (SSRs), also known as microsatellites and short tandem repeats (STRs), are specific DNA sequences commonly found in eukaryotic genomes, consisting of tandemly repeated units of one to six nucleotides flanked by non-repetitive regions. These repetitions generally arise due to slipped-strand mispairing and resultant error(s) during DNA replication, repair, or recombination [7]. SSRs have been developed into one of the most widely used genetic markers due to their high abundance, genome-wide coverage, presence of multiple alleles with variability in the number of repeats among individuals, codominant inheritance, and high reproducibility [8].

SSRs have been widely used to study genetic variability and population structure in a wide array of organisms, including parasites and vectors of infectious diseases such as leishmaniasis [8]. In earlier studies, SSR markers were identified using enrichment methods because sand fly genome sequences were unavailable [9, 10]. However, genome sequences of *Phlebotomus papatasi* and *Lutzomyia* species are now available. Massive advancements in sequencing technologies have led to a rapid increase in the availability of various types of DNA sequence data. With genomic and expressed sequence tag (EST) datasets becoming available, labor-intensive methods for generating SSR markers, such as enrichment methods, have been gradually replaced by *in silico* mining of SSRs [8, 11–15].

*In silico* mining of SSRs involves identifying microsatellite motifs within genome assemblies using computational tools. Several automated, user-friendly programs are now available for this purpose, enabling efficient detection of SSRs in large genomic datasets. The choice of software typically depends on the research objectives, the type of genome data, and the desired motif parameters [16]. This approach allows rapid, cost-effective, and high-throughput discovery of potential SSR markers compared with traditional laboratory screening methods. However, despite the availability of the *P. argentipes* genome at the scaffold level, no comprehensive analysis of its SSR content has been reported to date. This lack of information has limited the development of species-specific molecular markers for population genetic studies.

Therefore, the present study aimed to expand genomic resources for *P. argentipes* through *in silico* mining and characterization of perfect SSRs from a scaffold-level genome assembly, followed by field validation of selected markers across two *P. argentipes* populations in Sri Lanka to evaluate polymorphism and their utility as reliable genotyping tools. To the best of our knowledge, this represents the first genome-wide development and experimental validation of SSR markers in *P. argentipes*. The validated markers provide valuable genomic resources for future studies on gene flow, genetic diversity, and population structure, which are critical for understanding the epidemiology of leishmaniasis and supporting targeted vector control strategies in regions where *P. argentipes* serves as the principal vector.

## Results

### Prevalence and length variation of different types of SSRs

**Figure 1:**
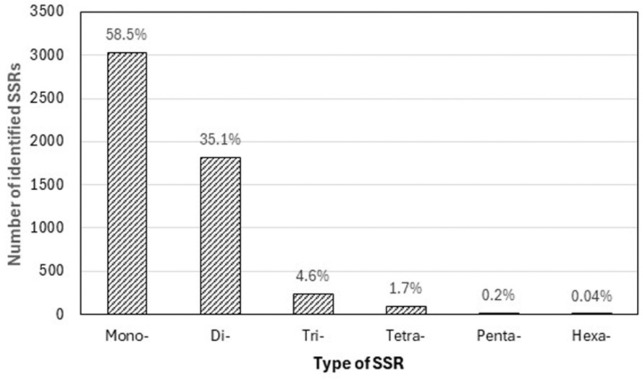
Frequency distribution of simple sequence repeat (SSR) types identified in the *Phlebotomus argentipes* genome. Mononucleotide and dinucleotide repeats dominate the SSR landscape, while higher-order repeat motifs occur at low frequencies. Percentages denote the relative abundance of each SSR class.

Out of the 63 scaffolds analyzed, 5173 SSRs were identified and characterized by repeat type and structure (Supplementary Table S1). Mononucleotide motifs were the most abundant, accounting for 58.5% of the total SSRs characterized, followed by dinucleotides (35%), trinucleotides (4.6%), tetranucleotides (1.7%), pentanucleotides (0.16%) and hexanucleotides (0.04%) (Supplementary Table S2, Fig. 1). The distribution of lengths for all SSRs demonstrated that the occurrence of repeats declines exponentially with increasing repeat length (Fig. 1).

The dinucleotide repeats were the most prevalent among sequences exceeding 40 nucleotides in length, followed by trinucleotide repeats, while there were no instances of mono-, tetra-, penta-, or hexanucleotide repeats (Supplementary Table S2). The longest array length varied in different types of SSRs (Supplementary Table S3). The maximum array length was recorded for di-SSRs (104 nucleotides), followed by tri-SSRs (87 nucleotides), tetra-SSRs (36 nucleotides), and penta- and hexa-SSRs (30 nucleotides each), with the least for mono-SSRs (23 nucleotides).

### SSR motif types and their prevalence

The A/T motif class represented the majority of mononucleotides with 2788 SSR motifs (92.1%), while the G/C motif class included only 239 SSR motifs (7.9%). Among the 1,813 dinucleotide motifs identified, the AG/TC/CT/GA type was the most abundant (63.3%), followed by AC/TG/CA/GT (24%) and AT/TA (12.63%). No CG/GC repeats were detected in the genome of *P. argentipes* according to the set criteria in the two SSR identification tools (Supplementary Table S4)

For trinucleotide SSRs, 237 motifs and 8 motif classes were identified (Supplementary Table S5). The AAT/ATA/TAA/TTA/TAT/ATT motif class was the most abundant which accounted for 33.3%.

For tetranucleotide SSRs, 86 motifs and 16 motif classes were identified. The AAAG/TTTC/AGAA/TCTT/GAAA/CTTT/AAGA motif was the most abundant, accounting for 29%, followed by AAAT/TTTA/ AATA/TTAT/TATT/ATTT (21%), while other motifs were at lower frequencies (Supplementary Table S6).

For pentanucleotide SSRs, 8 motif classes were identified: AATAG, AATTG, AGAAA, ATTAG, GAGAA, GTTTA, TAAAT, TCGAA, which occurred at a frequency of one. Two identical hexanucleotide repeats with the motif TGGGGG were present in the genome.

### Experimental validation and polymorphism assessment of SSR markers

A total of 22 microsatellite primers designed through *in silico* mining were initially screened for amplification using genomic DNA extracted from *P. argentipes*. All 22 primer pairs produced clear and reproducible PCR amplicons, confirming successful amplification. Bidirectional sequencing of representative amplicons verified the presence of expected SSR motifs and repeat structures. Based on amplification consistency and clarity of PCR products, 11 SSR loci were selected for fluorescent labeling and further polymorphism analysis. Fragment analysis across two geographic populations [Ambalantota/Geo 1(6.154590° N, 81.006640° E) and Polpithigama/Geo 2 (7.84303° N, 80.39395° E) Sri Lanka] revealed successful amplification across multiple isolates. Eleven SSR loci exhibited polymorphism and were retained for population genetic evaluation (Table 1).

The number of alleles per locus (Na) varied from 1 (IC08 in Geo 2) to 8 (IC27 in Geo 2), indicating moderate to high allelic diversity across loci and populations. Observed heterozygosity (Ho) ranged from 0.00 (IC08) to 0.75 (IC21), while expected heterozygosity (He) ranged from 0.29 (IC75) to 0.85 (IC27), demonstrating substantial genetic variability within populations. Loci IC27, IC51, IC68, and IC74 consistently exhibited high genetic diversity, with He values exceeding 0.80 (Table 1).

Polymorphism information content (PIC) values ranged from 0.26 (IC75) to 0.81 (IC27), indicating variable marker informativeness. Based on PIC classification, one locus was lowly polymorphic, one was moderately polymorphic, three were highly polymorphic, and four loci (IC27, IC51, IC68, and IC74) were very highly polymorphic, confirming their strong discriminatory power for population genetic analysis.

Population structure analysis revealed low to moderate genetic differentiation between Ambalantota and Polpithigama populations, with F_ST_ values ranging from 0.03 to 0.10, indicating limited but detectable genetic structuring and suggesting ongoing gene flow between the two geographic locations. The overall fixation index (F_IT_) ranged from approximately 0.05 to 0.45 across loci, indicating variable levels of total heterozygote deficit including both within- and between-population effects. Most loci showed positive F_IT_ values, reflecting a general reduction in heterozygosity relative to Hardy-Weinberg expectations, while locus IC21 showed negative deviation consistent with heterozygote excess (Table 1).

## Discussion

Population genetic studies of *P. argentipes* have been constrained by the limited availability of validated molecular markers [8]. By integrating *in silico* SSR mining with experimental wet-lab validation, the present study addresses this gap and provides a new panel of polymorphic microsatellite markers for *P. argentipes*. The genome-wide abundance and diversity of SSRs identified from the scaffold-level genome assembly are consistent with the recognized utility of SSRs as informative markers for population genetic analyses in sand flies [10, 21]. Furthermore, the successful amplification, sequence confirmation, and high polymorphism observed at several loci, reflected by elevated heterozygosity and polymorphism information content values, highlight the suitability of these markers for fine-scale population genetic studies. Such SSR markers are essential for investigating population structure, gene flow, and dispersal in *P. argentipes*, which are key determinants of vector dynamics and *Leishmania* transmission patterns [22, 23].

The *in silico* SSR mining in this study was based on the scaffold-level genome assembly of *P. argentipes*, which currently represents the most comprehensive genomic resource available for this species in the NCBI database [17]. The high assembly quality and contiguity, as reflected in favorable scaffold N50 and L50 values, provide confidence in the accuracy of SSR identification by minimizing gaps and assembly artefacts. The successful application of scaffold-level genome assemblies for reliable SSR mining has also been demonstrated in other dipteran insects, including multiple mosquito species and *Drosophila melanogaster* [24], supporting the robustness of the approach adopted here.

Previous population genetic studies of *P. argentipes* using mitochondrial markers such as *COI*, *Cyt b*, *ND4*, and ITS2 in Sri Lanka and India have reported limited resolution of fine-scale genetic differentiation and weak population structure, suggesting extensive gene flow among geographical populations when assessed using these loci [3, 25–27]. Similarly, *COI*-based barcoding of *P. argentipes* in Nepal enabled reliable species identification and broad haplotype comparisons but lacked the resolution required for detailed population genetic inference [28]. Collectively, these findings from the Indian subcontinent highlight the limitations of mitochondrial markers in resolving fine-scale population structure and underscore the need for highly polymorphic nuclear markers such as SSRs for comprehensive population genetic analyses.

The frequency and length distribution of SSRs in the *P. argentipes* genome mirror those patterns commonly observed in eukaryotic genomes. Using stringent repeat-length thresholds optimized for identifying polymorphic loci, the present study revealed a predominance of shorter SSR arrays, with repeat frequency declining exponentially as array length increased. This trend is consistent with the inherent instability of longer repeat tracts, which experience higher mutation rates and reduced evolutionary persistence [29–32]. Similar length-dependent distributions have been reported across diverse insect and eukaryotic genomes [24, 29, 33, 34], supporting the appropriateness of the applied criteria.

Motif composition analysis revealed a strong bias toward A/T-rich SSRs in the *P. argentipes* genome. Mononucleotide repeats were dominated by polyA/polyT motifs, accounting for 92.1% of all mononucleotide SSRs, whereas polyG/polyC motifs were rare (7.9%). This pronounced A/T bias is consistent with observations in humans, *Drosophila*, *C. elegans*, *Arabidopsis*, yeast, and many mosquito species [24, 29], as well as in Asian and Western honey bees, Rhesus monkeys, bovids, and birds [15, 35, 36]. The predominance of polyA/polyT repeats has been attributed to mutational processes or transposition events involving poly-A tracts [37, 38].

Among dinucleotide repeats, the AG/TC/CT/GA motif class was the most abundant in *P. argentipes*, comprising 63.3% of all dinucleotide SSRs, followed by AC/TG/CA/GT (24.1%) and AT/TA (12.6%). No perfect CG/GC repeats meeting the selection criteria were identified. A similar dominance of AG-type dinucleotides and scarcity of CG repeats has been reported in EST-derived SSRs of *P. papatasi* [8] and across a wide range of eukaryotic genomes, including insects and vertebrates [8, 15, 24, 29]. While CpG depletion in vertebrates is often linked to cytosine methylation and subsequent deamination [39], cytosine methylation patterns vary widely among insects, and data on CpG methylation in phlebotomine sand flies remain limited [40, 41]. Therefore, the absence of (CG)n repeats in *P. argentipes* cannot be conclusively attributed to CpG suppression.

Trinucleotide SSRs were dominated by AAT-class motifs, accounting for 33.3% of all trinucleotide repeats. This pattern closely mirrors earlier findings in phlebotomine sand flies, where AAT-class repeats were reported to be highly abundant in *P. papatasi*, *P. langeroni*, *Lutzomyia whitmani*, and *L. longipalpis* [42]. Genome-wide studies across eukaryotes, including *Drosophila*, mammals, plants, and yeast, have similarly reported a predominance of AT-rich trinucleotide motifs, particularly AAT, reflecting overall base composition biases in eukaryotic genomes [43, 44]. Importantly, AAT-class trinucleotides are more frequently located in introns and untranslated regions than in exons, reducing their likelihood of functional constraint and supporting their utility as neutral markers for population genetic studies [42].

Tetranucleotide repeats in *P. argentipes* were predominantly AAAG- and AAAT-class motifs, consistent with findings in *P. papatasi*, multiple mosquito species, and *Drosophila* [8, 23, 26]. As observed in other insects, penta- and hexanucleotide SSRs were rare [23], with only a single AATTG pentanucleotide motif detected, consistent with the rare occurrences reported for *P. papatasi* ESTs [8].

Together, these findings indicate that although microsatellite composition and abundance vary among major taxonomic groups, there is a strong conservation within insects, likely reflecting shared mutational mechanisms or selective constraints [45]. Beyond their utility as molecular markers, factors influencing microsatellite variation are also biologically relevant as mutations [46].

Through integrated *in silico* mining, PCR screening, and sequencing confirmation, we demonstrate that all 22 loci successfully amplified and contained authentic SSR motifs. However, comparison between *in silico* predictions and *in vitro* validation revealed that, for several loci, the observed repeat lengths were shorter than those predicted computationally, highlighting potential discrepancies due to sequence assembly limitations or natural variation. Of the 22 loci, a subset of 11 markers exhibited clear and reproducible polymorphism and were selected for downstream validation using DNA extracted from individual sand flies. During this process, it was observed that a minimum DNA concentration of approximately 30 ng/μL was required to achieve consistent and reliable amplification. Fragment analysis across two geographic populations in Sri Lanka revealed moderate to high allelic diversity, with several loci (IC27, IC51, IC68, and IC74) showing consistently high expected heterozygosity and polymorphic information content, highlighting their strong discriminatory power. Population genetic analysis further indicated low to moderate genetic differentiation between Ambalantota and Polpithigama, suggesting limited but detectable population structuring and ongoing gene flow. In addition, variation in fixation indices reflected locus-specific deviations from Hardy–Weinberg equilibrium, with a general trend toward heterozygote deficiency and occasional heterozygote excess. Collectively, these results confirm that the validated SSR panel is robust, informative, and suitable for investigating genetic diversity, population structure, and gene flow in *P. argentipes*, thereby providing a valuable genomic resource for understanding vector population dynamics and supporting leishmaniasis control strategies.

In conclusion, this study provides the first genome-wide development and experimental validation of SSR markers in *P. argentipes*, addressing a critical gap in available nuclear markers for this important leishmaniasis vector. The validated SSR panel demonstrated high amplification success, substantial polymorphism, and strong discriminatory power, with several loci exhibiting high heterozygosity and PIC values. Population genetic analyses revealed low to moderate genetic differentiation between Sri Lankan populations, indicating ongoing gene flow but detectable structuring. Overall, these markers provide a robust and informative toolset for future investigations of genetic diversity, population structure, and transmission dynamics of *P. argentipes*, with direct relevance for improving vector control and understanding leishmaniasis epidemiology.

## Methods

### In silico Identification of SSRs in the Phlebotomus argentipes Genome

**Figure 2:**
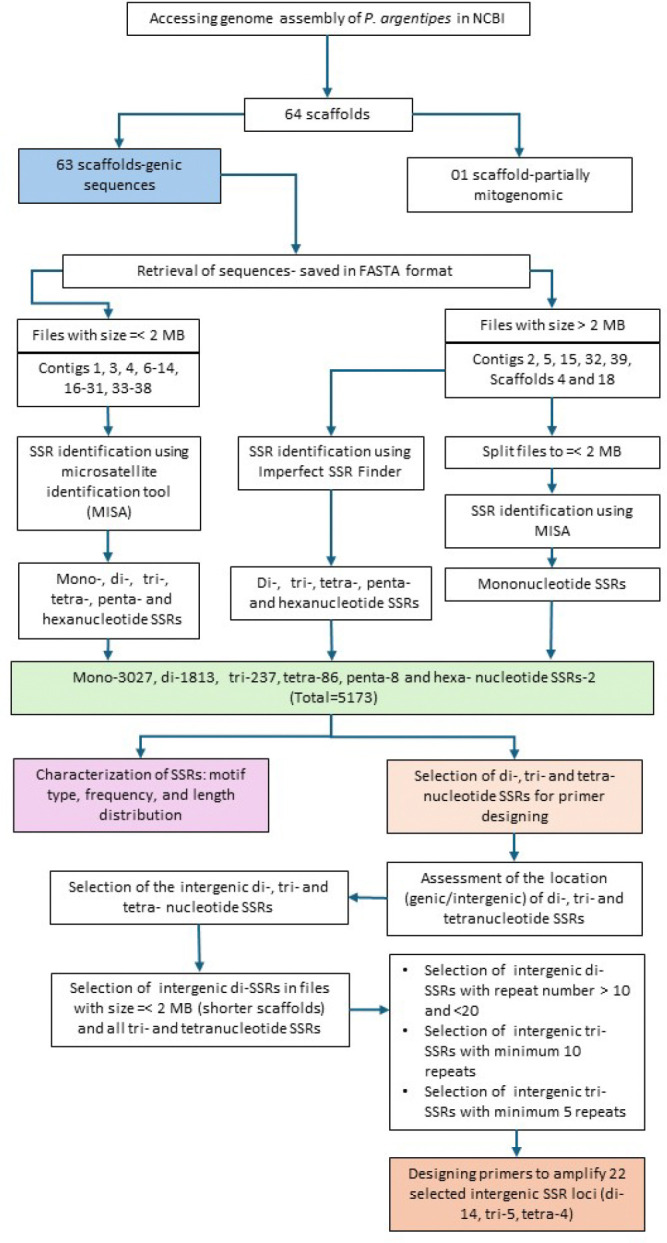
Genomic distribution of SSR loci selected for primer design in *Phlebotomus argentipes*. SSRs were mined from 64 genomic scaffolds, and intergenic SSR loci were selected for primer design. Twenty-two SSR primer pairs were successfully designed and distributed across multiple scaffolds for subsequent experimental validation.

The strategy adopted for the identification and selection of simple sequence repeats (SSRs) in *P. argentipes* is summarized in Fig. 2. The scaffold-level genome assembly of *P. argentipes* (96.7 Mb; accession GCF_947086385.1) was retrieved from the NCBI database (https://www.ncbi.nlm.nih.gov/datasets/genome/GCF_947086385.1/) [17] and comprised 64 scaffolds, including 63 genomic scaffolds and one partial mitochondrial genome sequence. The 63 genomic sequences were downloaded in FASTA format for SSR analysis. Although the assembly is scaffold-based, the first 61 sequences are annotated as contigs in the NCBI database, and this annotation was retained in the present study. Based on file size constraints of the SSR detection tools, the sequences were categorized into those smaller or larger than 2 MB, with seven sequences (contigs 2, 5, 15, 32 and 39, and scaffolds 4 and 18) classified as large sequences. Perfect SSRs in sequences smaller than 2 MB were identified using the Microsatellite Identification Tool (MISA; https://webblast.ipk-gatersleben.de/misa/) [18], applying thresholds of ≥ 10 repeats for mono- and dinucleotide motifs, ≥ 7 repeats for trinucleotide motifs, and ≥ 5 repeats for tetra-, penta- and hexanucleotide motifs. SSRs in sequences larger than 2 MB were identified using the Imperfect SSR Finder of the Agricultural Research Service, United States Department of Agriculture (https://ssr.nwisrl.ars.usda.gov/) [19], with default settings and the same repeat thresholds. As this tool does not detect mononucleotide repeats, large sequences were split into fragments smaller than 2 MB using an online text file splitter (https://textfilesplitter.com/), analyzed using MISA, and the identified mononucleotide SSRs were compiled.

### Characterization of SSRs

The identified SSRs were characterized in terms of their type and frequency within the 63 analyzed contigs. The density (number of SSRs per Mb of genomic sequence) and abundance (proportion of a given type of SSR among total SSRs) were used to measure the frequency of SSRs.

The SSR motif class types were defined as follows. In SSR analysis, repeat motifs were considered equivalent if they appeared in different reading frames or on the complementary DNA strand. For example, a polyA repeat is treated as identical to a polyT repeat on the complementary strand. Similarly, a dinucleotide repeat such as (AC)_n_ was regarded as equivalent to (CA)_n_, (TG)_n_, and (GT)_n_ because the sequence remains the same when read in reverse or on the complementary strand. The same principle was applied to trinucleotide repeats. A repeat like (AGC)_n_ was considered identical to (GCA)_n_, (CAG)_n_, (CTG)_n_, (TGC)_n_, and (GCT)_n_, as all these variations represent the same sequence when viewed in different reading frames and on the complementary strand. Due to this classification system, the number of unique microsatellites repeat classes is reduced. Specifically, mononucleotide repeats have only two unique classes (e.g., polyA/polyT and polyC/polyG), while dinucleotide repeats have four distinct classes. Trinucleotide repeats are grouped into 10 unique classes, and tetranucleotide repeats are categorized into 33 distinct groups [20]. This approach simplifies microsatellite analysis by grouping functionally identical sequences together, reducing redundancy in classification. Additionally, the frequencies of SSRs belonging to ten classes of nucleotide array lengths were analyzed to study the length variation among different types of SSRs. Furthermore, SSR motifs with the longest array length, belonging to the different types of SSRs, were compared.

### Selection of SSRs and primer designing

The genomic location of all identified di-, tri- and tetranucleotide SSRs was determined using the genome annotation of *P. argentipes* generated by the NCBI Gnomon gene prediction pipeline (https://www.ncbi.nlm.nih.gov/datasets/genome/GCF947086385.1/) [17]. Annotation data were downloaded in General Feature Format (GFF3), and SSR coordinates obtained from MISA and the Imperfect SSR Finder were compared with annotated gene features (exons and introns) to classify SSRs as genic or intergenic. This comparison was performed using a custom Excel-based macro to facilitate filtering and extraction of SSR positions. Only intergenic SSRs were retained for downstream analysis, as they are expected to exhibit higher polymorphism and are therefore more suitable for population genetic studies. Dinucleotide SSRs with 10–20 repeat units identified from sequences smaller than 2 MB were selected to ensure broad scaffold coverage, while trinucleotide SSRs with ≥ 10 repeats and tetranucleotide SSRs with ≥ 5 repeats from sequences of both size categories were included. Primers flanking the selected intergenic SSRs were designed using Primer3Plus, applying standard criteria for primer length (18–24 bp), melting temperature (59–61°C), GC content (48–52%), and amplicon size (150–250 bp), with adjustments made for AT-rich regions when necessary. Primer specificity and secondary structures were evaluated using OligoAnalyzer^™^ (IDT), and only primers without significant hairpins or primer–dimer formation (ΔG > − 9 kcal/mol) were retained. In total, 22 primer pairs were successfully designed (Table 2) from SSR loci distributed across 63 genomic scaffolds for experimental validation.

### Laboratory validation and polymorphism analysis

All 22 SSR primers designed *in silico* (Table 2) were initially validated under optimized PCR conditions using genomic DNA extracted from individual laboratory-reared *P. argentipes* adults, with one sand fly used per primer (QIAamp DNA Micro Kit, Qiagen, Germany). Amplicons were visualized on 2% agarose gels, and all 22 primers produced clear and specific products. These PCR products were subsequently sequenced bidirectionally, and the resulting sequences were analyzed using MISA to confirm the presence of SSR motifs, repeat number, and repeat diversity.

Based on amplification success, 11 SSR primers that produced clear and reproducible PCR products were selected for fluorescent labeling and further analysis (Table 1). These 11 primers were further validated under optimized PCR conditions using genomic DNA extracted from individual laboratory-reared *P. argentipes* adults, with one sand fly used per reaction. Subsequently, field validation was performed using genomic DNA isolated from individual sand flies collected from two geographic locations in Sri Lanka: Ambalantota (Southern Province) and Polpithigama (North Western Province). A total of 20 sand flies (10 individuals per location) were analyzed, with each sample tested individually using 11 SSR markers. The forward primers of these loci were fluorescently labeled with 6-FAM or HEX dyes. PCR amplification was performed in a 20 μL reaction containing 1× PCR buffer, 2 mM MgCl₂, 0.2 mM dNTPs, 0.4 μM of each primer, 1 U *Taq* DNA polymerase, and 5–10 ng template DNA. The thermocycling conditions were as follows: initial denaturation at 94°C for 5 min; 35 cycles of 94°C for 30 s, locus-specific annealing at 49–60°C for 30 s, and 72°C for 30 s; followed by a final extension at 72°C for 5 min.

**Figure 3:**
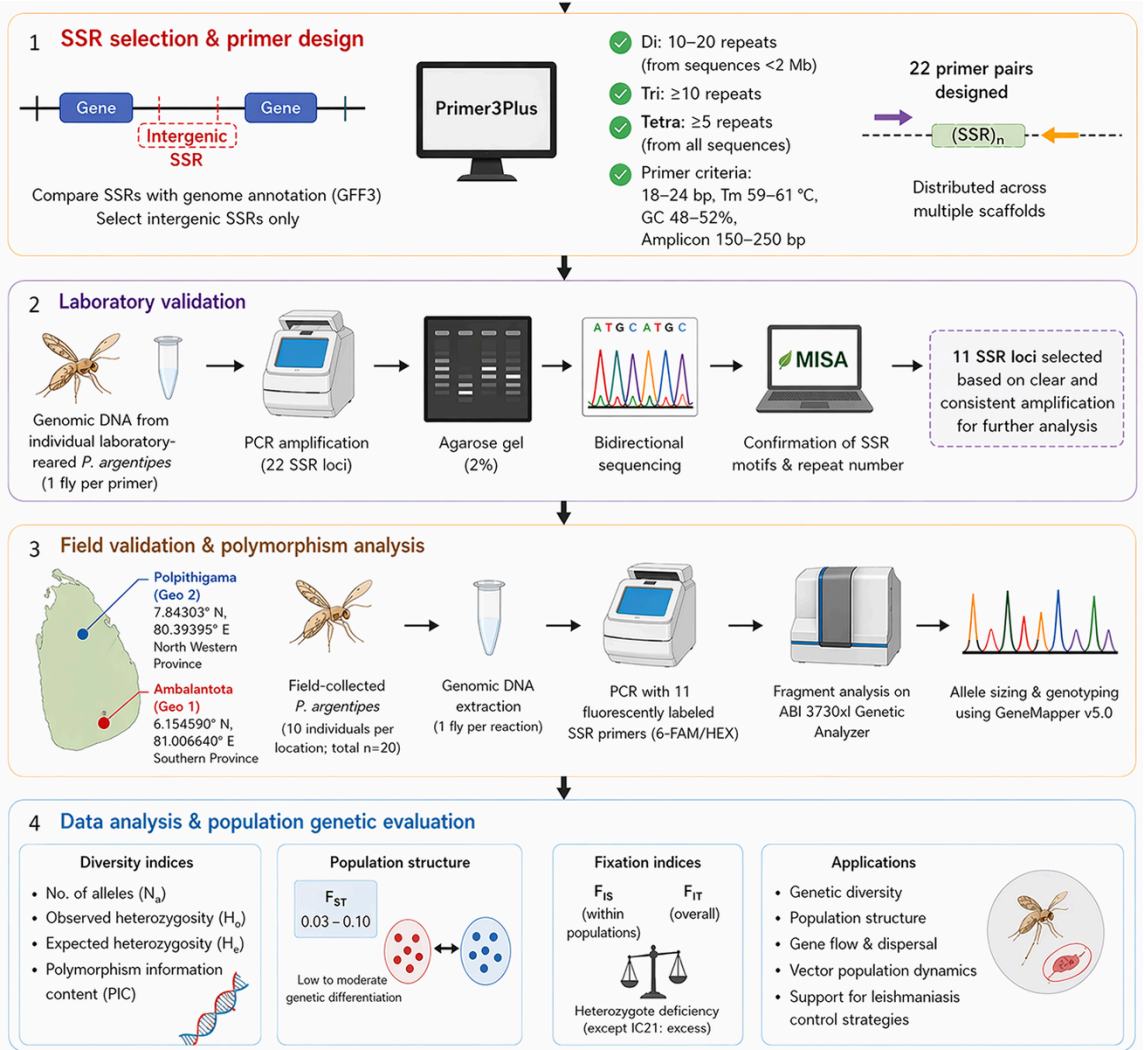
Schematic illustration of the workflow for identification, validation, and population genetic analysis of SSR markers in *Phlebotomus argentipes*.

Fragment analysis of fluorescently labeled PCR products was performed on an ABI 3730xl Genetic Analyzer using GeneScan^™^ 500 LIZ^™^ as the internal size standard. Allele sizing and polymorphism assessment were conducted using GeneMapper v5.0. The overall workflow has been demonstrated in Fig. 3.

### Data Analysis

Microsatellite genotyping was performed by Macrogen Inc. (Seoul, Republic of Korea) using capillary electrophoresis–based fragment analysis. Allele sizes were determined from electropherograms using Peak Scanner software. Repeat motifs and fragment length distributions were assessed based on the expected SSR repeat structure of each locus. Descriptive statistics, including the number of alleles and allele size range per locus, were calculated. Marker polymorphism was evaluated by estimating polymorphism information content (PIC) as well as observed (Ho) and expected heterozygosity (He) to assess the suitability of the SSR markers for population genetic analyses.

## Supplementary Material

Supplementary Files

This is a list of supplementary files associated with this preprint. Click to download.

• Table1.docx

• Table2.docx

• SupplementaryTableS2.docx

• SupplementaryTableS6.docx

• SupplementaryTableS1.docx

• SupplementaryTableS5.docx

• SupplementaryTableS3.docx

• SupplementaryTableS4.docx

• SupplementaryFileSSRsequencedata.pdf

## Figures and Tables

**Table 1:** Polymorphism characteristics of experimentally validated SSR markers in *Phlebotomus argentipes* isolate in two geographical locations: Ambalantota (Geo location 1) and Polpithigama (Geo location 2), Sri Lanka

Locus name	NCBI Accession	No. of alleles (Geo 1)	No. of alleles (Geo 2)	Ho	He	F_IS_	F_ST_	PIC
IC07	PZ269930	7	6	0.43	0.73	0.41	0.05	0.69
IC02	PZ269931	7	7	0.45	0.72	0.38	0.03	0.68
IS18	PZ269932	4	5	0.40	0.58	0.31	0.06	0.52
IC75	PZ269933	4	4	0.20	0.29	0.31	0.04	0.26
IC08	PZ269934	2	1	0.00	0.44	1.00	0.10	0.33
IC21	PZ269935	4	3	0.75	0.66	-0.14	0.05	0.61
IC27	PZ269936	6	8	0.60	0.85	0.29	0.04	0.81
IC50	PZ269937	3	4	0.50	0.63	0.21	0.05	0.57
IC51	PZ269938	6	7	0.57	0.82	0.30	0.04	0.79
IC68	PZ269939	4	5	0.67	0.83	0.19	0.05	0.81
IC74	PZ269940	4	5	0.70	0.78	0.10	0.04	0.75

H_0_: Observed heterozygosity, H_e_: Expected heterozygosity, F_IS_: Fixation Index within subpopulations, FIT: Fixation Index among subpopulations, PIC: Polymorphism information content

**Table 2:** Primers designed to amplify the selected SSRs, including the SSR, product size, forward and reverse primer sequences and annealing temperature (T_m_).

No.	Scaffold/Contig	Primer sequence (5'–3')	Repeat motifs found in *in silico* mining(n)	Product size (bp)	Repeat motifs found in *in vitro* PCRs (n)	PCR annealing T/°C
1	IC03	F-CCCGAGTGCCACAAGTAAAT R-GTTGAGGCTGTGGTGAATGA	(TAA)11	238	(TAA)11	56.1
2	IC07	F-CTTATGATTTGCCCCTTG R-GTGTGGGAATTGATTACACC	(GAG)12	179	(GAG)12	49.0
3	IC02	F-ATCAGCCTCTTGCCATGAAC R-GTGAAATCCACACGACGATG	(CTC)10	158	(CTC)4	52.2
4	IC15	F-AAAGTACGTGTGGAAGGGAGTG R-GGCACAAACACACAGTCGAA	(GAA)12	156	(GAA)9	57.3
5	IS18	F-GGAAGATCTGGGTGATTGGA R-GAGATTGTCTTGCAGCCTCA	(GGA)20	156	(GGA)17	52.0
6	IC20	F-TACTGCATCAGCAAAATCAG R-CCGTTAAGAAAAGGTATACGG	(TTTA)7	198	(TTTA)7	56.2
7	IC75	F-GTTCACCGATGTCTGAGTGTTG R-TTTCCGCTCCCCAGTACTTA	(TAGA)6	153	(TAGA)6	55.2
8	IC75	F-TCCGTCTCGACAATACAGCA R-AGATCGCAATACGGCAGCTA	(ATTC)5	190	(ATTC)4	53.1
9	IC8	F-CTCTCCGCATCTTCCATCTT R-CGGCAATGTAGGGGAAAA	(GT)15	237	(GT)11	51.4
10	IC8	F-GTGGAAAGCGTGAAAACTCC R-AACTTTCAACCCTCCCTTCC	(TG)10	191	(TG)7	58.2
11	IC8	F-AGCTCTGCTCGGCTTTTCTT R-CAATGGATTCTCGGCTGTCT	(TG)10	180	(TG)10	54.0
12	IC10	F-CCTCACTCTTTTCGCTCCAA R-TTGTCTGCTTGCTTGTCTGG	(AG)12	165	(AG)12	60.4
13	IC17	F-ATCTTGGCTTCTGCTCTGGA R-CATCGCATTTGTGGGTTG	(TC)18	203	(TC)15	47.0
14	IC21	F-CTGTCTCAAATCCACCGACA R-TTATGCCAGCACGGCTTT	(AG)10	182	(AG)10	56.3
15	IC21	F-GACGACGATGGGCAAATAA R-GCGAGACGTGCAGAGCAATTA	(TG)10	175	(TG)9	54.2
16	IC27	F-TCATGGGCGAAAGGGTAA R-ACGTGATGCTGAGGATTGTG	(CA)18	197	(CA)6	54.0
17	IC46	F-CGTCATGGTGGCAAATCA R-AACAACGGCTCAACAGTCCT	(CT)13	152	(CT)8	51.0
18	IC50	F-CCTGCATCCAGGACATTTCT R-GGGACATTGATGGAAAGTGG	(CT)11	158	(CT)8	54.3
19	IC51	F-TTGCACCCAAGTTGACCA R-CAAAAGCTCTGGCTGTGTTG	(TC)11	198	(AG)9	54.2
20	IC66	F-TTTCTCCACCTCTCGTTTCG R-TCCATCTCGCTCATCTTGTG	(GA)11	210	(GA)7	59.6
21	IC68	F-GTGATGCAAGCCTCTGAT R-TAGGAAATGGGTGAGAGGACGAA	(GT)11	161	(GT)5	54.4
22	IC74	F-AAGATCCCGAAAGAAGGAT R-CACGACAAATTGCATGTCTA	(TC)16	176	(TC)14	49.0

**Table 2:** Primers designed to amplify the selected SSRs, including the SSR, product size, forward and reverse primer sequences and annealing temperature (°C).

No.	Scaffold/Contig	Primer sequence (5'–3')	Repeat motifs found in *in silico* mining(n)	Product size (bp)	Repeat motifs found in *in vitro* PCRs (n)	PCR annealing T/°C
1	IC03	F-CCCGAGTGCCACAAGTAAAT R-GTTGAGGCTGTGGTGAATGA	(TAA)11	238	(TAA)11	56.1
2	IC07	F-CTTATGATTTGCCCCTTG R-GTGTGGGAATTGATTACACC	(GAG)12	179	(GAG)12	49.0
3	IC02	F-ATCAGCCTCTTGCCATGAAC R-GTGAAATCCACACGACGATG	(CTC)10	158	(CTC)4	52.2
4	IC15	F-AAAGTACGTGTGGAAGGGAGTG R-GGCACAAACACACAGTCGAA	(GAA)12	156	(GAA)9	57.3
5	IS18	F-GGAAGATCTGGGTGATTGGA R-GAGATTGTCTTGCAGCCTCA	(GGA)20	156	(GGA)17	52.0
6	IC20	F-TACTGCATCAGCAAAATCAG R-CCGTTAAGAAAAGGTATACGG	(TTTA)7	198	(TTTA)7	56.2
7	IC75	F-GTTCACCGATGTCTGAGTGTTG R-TTTCCGCTCCCCAGTACTTA	(TAGA)6	153	(TAGA)6	55.2
8	IC75	F-TCCGTCTCGACAATACAGCA R-AGATCGCAATACGGCAGCTA	(ATTC)5	190	(ATTC)4	53.1
9	IC8	F-CTCTCCGCATCTTCCATCTT R-CGGCAATGTAGGGGAAAA	(GT)15	237	(GT)11	51.4
10	IC8	F-GTGGAAAGCGTGAAAACTCC R-AACTTTCAACCCTCCCTTCC	(TG)10	191	(TG)7	58.2
11	IC8	F-AGCTCTGCTCGGCTTTTCTT R-CAATGGATTCTCGGCTGTCT	(TG)10	180	(TG)10	54.0
12	IC10	F-CCTCACTCTTTTCGCTCCAA R-TTGTCTGCTTGCTTGTCTGG	(AG)12	165	(AG)12	60.4
13	IC17	F-ATCTTGGCTTCTGCTCTGGA R-CATCGCATTTGTGGGTTG	(TC)18	203	(TC)15	47.0
14	IC21	F-CTGTCTCAAATCCACCGACA R-TTATGCCAGCACGGCTTT	(AG)10	182	(AG)10	56.3
15	IC21	F-GACGACGATGGGCAAATAA R-GCGAGACGTGCAGAGCAATTA	(TG)10	175	(TG)9	54.2
16	IC27	F-TCATGGGCGAAAGGGTAA R-ACGTGATGCTGAGGATTGTG	(CA)18	197	(CA)6	54.0
17	IC46	F-CGTCATGGTGGCAAATCA R-AACAACGGCTCAACAGTCCT	(CT)13	152	(CT)8	51.0
18	IC50	F-CCTGCATCCAGGACATTTCT R-GGGACATTGATGGAAAGTGG	(CT)11	158	(CT)8	54.3
19	IC51	F-TTGCACCCAAGTTGACCA R-CAAAAGCTCTGGCTGTGTTG	(TC)11	198	(AG)9	54.2
20	IC66	F-TTTCTCCACCTCTCGTTTCG R-TCCATCTCGCTCATCTTGTG	(GA)11	210	(GA)7	59.6
21	IC68	F-GTGATGCAAGCCTCTGAT R-TAGGAAATGGGTGAGAGGACGAA	(GT)11	161	(GT)5	54.4
22	IC74	F-AAGATCCCGAAAGAAGGAT R-CACGACAAATTGCATGTCTA	(TC)16	176	(TC)14	49.0

## Data Availability

The data supporting the conclusions of this study are included within the article and its supplementary materials. The nucleotide sequences generated in this study have been deposited in GenBank under accession numbers PZ269930–PZ269940. At the time of submission, these records are still undergoing processing following removal of the embargo and will be made publicly available upon completion of processing by NCBI. In the meantime, the full raw sequence dataset has been provided as a supplementary file to ensure immediate access for reviewers and readers.
